# Metabolomic Analysis of *Phytophthora parasitica* Growth in the Presence of β‐sitosterol Indicates Adaptive Mechanisms Modulated by Sterols

**DOI:** 10.1002/jobm.70149

**Published:** 2026-02-04

**Authors:** Pâmela Ponce Martins, Evandro Silva, Marcus Vinicius Fernandes Prior, João Marcos Martins Ferreira, Marina Erê Pimenta Santos, Flavia Rodrigues Alves Patrício, Taicia Pacheco Fill, Jorge Maurício Costa Mondego

**Affiliations:** ^1^ LABIPLAM, Centro de Recursos Genéticos Vegetais Instituto Agronômico (IAC) Campinas Brasil; ^2^ Pós‐Graduação em Genética e Biologia Molecular UNICAMP Campinas Brasil; ^3^ Laboratório de Biologia Química Microbiana Instituto de Química, UNICAMP Campinas Brasil; ^4^ Pós‐Graduação em Agricultura Tropical e Subtropical, IAC Campinas Brasil; ^5^ Instituto Biológico Laboratório de Fitopatologia Campinas Brasil

**Keywords:** metabolomics, *Phytophthora parasitica*, sterol adaptation, β‐sitosterol

## Abstract

*Phytophthora parasitica* is a generalist phytopathogenic oomycete that infects a series of crops with great economic interest, including tomato, tobacco, and citrus species. Sterols are essential lipids in eukaryotic organisms, playing a fundamental role in the structure and function of the cell membrane. Oomycete from Peronosporales order, including *Phytophthora* spp., are known for their inability to synthesize sterols, a characteristic that distinguishes them from many other organisms and influences their biology and pathogenicity. This dependence is not only crucial for zoospore sporulation but also for vegetative growth. Following the scenario at which sterol auxotrophy can interfere in the metabolism of such important phytopathogen, in this study we investigate whether β‐sitosterol modulates the metabolome of *P. parasitica* and its vegetative growth. For that, we used liquid chromatography–mass spectrometry LC‐HRMS combined with chemometric tools, to assess the metabolite profile of *P. parasitica* under different concentrations of β‐sitosterol in the culture media. Even though mycelial growth was not significantly affected by different sterol concentrations, an evident metabolic reprogramming of the oomycete was detected. Among the metabolites differentially produced in the sterol presence are the sphingolipid sphinganine, histidine and the nucleoside methylthioadenosine. We discuss the influence of these metabolites in vegetative growth of the oomycete and infer possible roles in environmental adaptation and pathogenicity.

**Abbreviations**
FBTs: folate‐biopterin transporters
GIPC: glycosyl inositol phosphoryl ceramides
GlcCer: glucosylceramide
GNPS: Global Natural Products Social Molecular Networking
LC‐HRMS: Liquid Chromatography‐High‐Resolution Mass Spectrometry
MTA: 5′‐methylthioadenosine
OSBPs: oxysterol‐binding proteins
PCA: Principal Component Analysis.
PTFE: polytetrafluoroethylene

## 1 Introduction

The oomycete *Phytophthora parasitica* (*Phytophthora nicotianae* pv. *parasitica*; *Peronosporales*) is a destructive pathogen with significant economic impact. This species has a broad host range and is known to cause devastating diseases in various crops, including tobacco, tomato, pepper, and ornamental plants [1]. In citrus crops, *P. parasitica* is particularly damaging, causing gummosis and root rot, which are diseases that pose major threats to citrus production worldwide [2, 3]. In Brazil, one of the world's largest producers of oranges and lemons, this oomycete can reduce productivity by more than 30% [4, 5], resulting in substantial economic losses and increased costs for disease management.

Reproduction, dispersal, and infection are fundamental processes for the success of *Phytophthora* as a phytopathogen. Asexual reproduction occurs via sporangia, which release biflagellate zoospores capable of moving in water in search of hosts [6]. This mechanism gives the pathogen a high dispersal capacity, especially in humid and irrigated regions. During infection, zoospores detect chemical signals exuded by plant roots, allowing directed navigation towards the plant [7]. Once on the root surface, zoospores encyst, germinate, and penetrate plant tissues, triggering host colonization. In addition to asexual reproduction, *Phytophthora* presents sexual reproduction via oogamy, forming resistant oospores that can remain viable in the soil for years, ensuring the persistence of the disease even in the absence of hosts [8].

Oomycetes of the order *Peronosporales*, including species of the genus *Phytophthora*, are notable for their inability to synthesize sterols de novo, an evolutionary trait that sets them apart from many other eukaryotic organisms, and profoundly impacts their biology and pathogenicity. These oomycetes have lost key genes required for the sterol biosynthesis pathway [9] and thus rely on exogenous sources to acquire these essential lipids [10]. Specifically, *Phythophthora* lacks the gene encoding squalene epoxidase (SqE), a pivotal enzyme catalyzing the epoxidation of squalene to 2,3‐oxidosqualene, which is a critical precursor in sterol biosynthesis [11, 12]. This sterol auxotrophy necessitates that *Phytophthora* species scavenge sterols from host tissues or the surrounding environment to support membrane integrity, growth, and infection processes. Consequently, sterol dependency represents a key aspect of *Phytophthora*'s pathogenic strategy and of its interactions within the plant–microbe interface. This dependence is crucial for vegetative growth and sporulation, as demonstrated in *P. cactorum* [13, 14] and *P. infestans* [15].

In fact, sterol biosynthesis inhibitors are the most important chemicals used to control plant diseases [16]. As the participation of sterols as players in plant–microbe interactions are becoming increasingly evident [17], we decided to assess the response of oomycetes to sterol different concentrations. In this study, we evaluate the compounds differentially produced in sterol depletion and abundance using untargeted metabolomics by LC‐HMRS, aiming to investigate the participation of sterol during mycelial growth of *P. parasitica*.

## 2 Materials and Methods

### 2.1 Cultivation of *Phytophthora parasitica* and Sterol Experiment

The cultivation of the oomycete was conducted at the Laboratory of Biotechnology and Plant‐Microorganism Interaction (LABIPLAM, Instituto Agronomico, Campinas), and at the Laboratory of Phytopathology (Instituto Biológico, Campinas). *Phytophthora parasitica* IAC01/95 was grown in Juice V‐8 agar medium containing 200 mL/L of V‐8 (20% v/v), 4.5 g/L of CaCO₃ (~0.045 mol/L), 17 g/L of agar (~1.7% w/v), and 0.4 g/L (~0,6 mM) of Pentabiotic Reforçado® Zoetis, Brazil; 6,000,000 IU, 8.5 g vial, (benzylpenicillinbenzatine, benzylpenicillinprocaine, benzylpenicillinpotassium, dihydrostreptomycin base ‐ sulfate, streptomycin base ‐ sulfate). The final volume was adjusted to 1 L with sterile distilled water as described by Romero and Gallegly [18].

β‐sitosterol (Sigma‐Aldrich, ≥ 70% purity) was initially diluted in chloroform (CHCl₃) at a ratio of 1 g per 1 mL, resulting in a 100% stock solution (1 g/mL). From this solution, three different concentrations were prepared for supplementation of the culture medium. First, 10 µL of the stock solution were added to 0.5 L of freshly autoclaved, still‐hot V8 medium to obtain a final concentration of 20 mg/L. Subsequently, 5 µL and 0.5 µL of the same stock were added to separate 0.5 L volumes of V8 medium to achieve final concentrations of 10 mg/L and 1 mg/L, respectively. In all conditions, β‐sitosterol was incorporated into the medium while it was still warm to ensure proper homogenization. The supplemented media were poured aseptically into sterile Petri dishes and allowed to solidify at room temperature. A control group was prepared using V8 medium without the addition of β‐sitosterol.

Description regarding the experiment of β‐sitosterol influence on the metabolites production of *P. parasitica* is described at results topics. The plates were kept in the dark at 27°C for a period of 10 days to allow mycelium growth. For each treatment, 10 replicates were performed, totaling 80 experimental units. Plates were checked macroscopically and microscopically to check possible oomycete phenotypic differences between treatments described above.

### 2.2 Untargeted Metabolomics Analysis

#### 2.2.1 Extraction and LC‐HRMS Analysis

After 10 days of *P. parasitica* growth, four plates from each treatment (total 32 plates) were randomly chosen for metabolomics analysis. The entire content of each culture plate, including the mycelium and medium, was cut into small pieces and immediately frozen in liquid nitrogen. The material was then ground into a fine powder using a mortar and pestle. A 100 mg aliquot of the resulting powder was transferred to a 2 mL microcentrifuge tube, and metabolites were extracted using 1 mL of methanol (MeOH), and left for 20 min in an ultrasonic bath at room temperature. The extracts were then centrifuged at 10,000 rpm for 10 min at 4°C. The resulting supernatants were collected, dried under a nitrogen stream, and resuspended in 1 mL of MeOH. Finally, the resuspended extracts were filtered through 0.22 μm PTFE syringe filters into 2 mL glass vials. Control plates containing only the culture medium (without fungal growth) were processed in parallel using the same extraction procedure to serve as blanks [19].

The samples were analyzed using a Thermo Fisher RSLCnano U3000 UHPLC instrument coupled with a Q‐Exactive Orbitrap‐MS spectrometer (Thermo Fisher Scientific, USA) equipped with a heated electrospray ionization (HESI) source. Chromatographic separation was performed with an Intensity Solo C18 2.2 µm column (2.1 mm x 100 mm, Bruker Daltonics, Bremen, Germany). Mobile phases were 0.1% (v/v) formic acid in H_2_O (A) and 0.1% formic acid in acetonitrile (B), 40°C for elution temperature and 250 µL/min for flow rate. The elution gradient consisted of 5% B (0–5 min), 40% B (5–10 min), 70% B (10–14 min), 80% B (14–15 min), 98% B (15–20 min) and 5% B (21–24 min). Mass spectra acquisition was performed using the following parameters: electrospray ionization in positive mode capillary voltage: equal to +3.5 kV; capillary temperature: 250°C, S‐lens of 50 V and *m/z* range 100–1500. LC‐MS/MS spectra were recorded using a Normalized Collision Energy (NCE) of 30 eV and five precursors per cycle were selected (data dependent acquisition – DDA ‐ mode) [19].

#### 2.2.2 Data Processing and Statistical Analyses

Raw data were converted using MSConvert software, generating files in mzXML format [20]. The converted files were processed using Mzmine version 3.9 [21]. The feature detection step was performed using a signal/noise value of 1.5 × 10^5^. The ADAP construction builder function was performed by the baseline cut‐off algorithm with a minimum group size of scans equal to 5 points, with a minimum intensity height of 1.5 × 10^5^ and a *m/z* tolerance of 0.01. MS^2^ scans were processed at a signal‐to‐noise ratio of 1.0 × 10^3^, with a tolerance of 0.01 for *m/z* values and 0.1 for retention time (RT) values. Isotopes were grouped using the “isotopic peak grouper” algorithm with a mass tolerance of 0.01 and RT 0.02 min. The peaks were aligned using the “join aligner.” The spectral data in.mgf format and the features quantification table based on the peak areas in.csv format were exported to the GNPS platform (https://gnps.ucsd.edu), using Feature Based Molecular Networking workflow.

### 2.3 Principal Component Analysis (PCA)

Principal Component Analysis (PCA) was performed in MetaboAnalyst (MetaboAnalyst 6.0, 2024) to reduce data dimensionality and identify patterns of variation among treatments. The variance explained by the principal components was analyzed to understand the contribution of each one to the total variability of the data.

### 2.4 Molecular Networking

Molecular networking analysis was performed using the Feature‐Based Molecular Networking (FBMN) workflow on the GNPS2 platform (https://gnps2.org), workflow version 2025.01.24. Input data consisted of.mgf files containing MS/MS spectra and a feature quantification table (.csv). Feature detection was carried out using Mzmine.

Network construction parameters were set as follows: fragment mass tolerance of 0.01 Da, precursor mass tolerance of 0.01 Da, and a maximum mass shift tolerance (analog search and networking max shift) of 1999 Da, with no analog search enabled. Spectral similarity was assessed using a cosine score threshold of 0.8, with a minimum of six matched peaks required for node connection. The network was built considering the top 10 nearest neighbors (topK = 10) for node connectivity, and the maximum topology component size was limited to 100 nodes. Window filtering (window_filter = 1) and active precursor filtering (precursor_filter = 1) were applied. No data normalization was performed.

Metabolite annotation was conducted by comparison against internal spectral libraries available on GNPS, using a best‐hit search strategy (library_topk = 1) with a minimum cosine score of 0.8 and at least six matched peaks. Results were exported for further visualization and analysis using dedicated molecular networking software [22].

### 2.5 Differential Metabolite Validation

Statistical analyses were conducted in the RStudio environment, using the R language. To assess the differences between treatments, the Wilcoxon signed‐rank test was applied, as it is a nonparametric approach appropriate for data that do not follow a normal distribution. Pairwise comparisons were performed between all treatments, considering all possible combinations. To account for multiple comparisons, p‐values were adjusted using the Bonferroni correction. Although this method is considered conservative, it was intentionally chosen to ensure that only the most consistent and biologically robust differences among treatments were retained as significant. This method was applied due to the moderate number of biological replicates (*n* = 4 per treatment), and a moderate dataset size (322 metabolites), which intrinsically diminish statistical power and increase the variability of *p*‐values. To minimize heteroscedasticity and bring the data distribution closer to normality, the values were previously transformed using a logarithm to base 10 [log₁₀(x)]. The significance level adopted was 5% (*p* < 0.05).

After applying the tests, the metabolites that presented a statistically significant difference between the SP and SP20 treatments and that, simultaneously, did not present a significant difference between SV20 and SP20 were selected. The selected metabolites were submitted to a new identity validation stage using the GNPS platform. The identity of the compounds was confirmed based on the spectral similarity between the experimentally acquired mass spectra and the reference spectra present in the GNPS libraries. For this validation, the cosine score was used as a similarity metric, with GNPS standard cutoffs typically set at 0.5 for preliminary matches and 0.7 for confident annotations. In our study, only metabolites with a cosine score equal to or greater than 0.8 were considered reliably annotated, adopting a stricter threshold to ensure higher confidence in metabolite identification. This value indicates a high degree of similarity between the experimental and reference spectra and is widely accepted as a criterion for identity attribution in mass spectrometry analyses [22].

Following spectral validation, a heatmap was generated in RStudio using the heatmap package to visualize the relative abundance of the selected metabolites across the experimental treatments. The data were standardized by row (z‐score transformation) to highlight relative differences in metabolite profiles. Additionally, bar plots were constructed to depict the quantitative behavior of all treatments for the three metabolites selected for discussion in this study. These plots were generated in RStudio using tidyverse, readxl, and scales packages, which enabled structured data manipulation, import, and visualization. The data were presented as a means intensity per treatment, with customized y‐axis scaling to facilitate interpretation and comparison across groups. These visualizations supported the interpretation of metabolomics variations observed among the analyzed treatments.

## 3 Results

**Figure 1:**
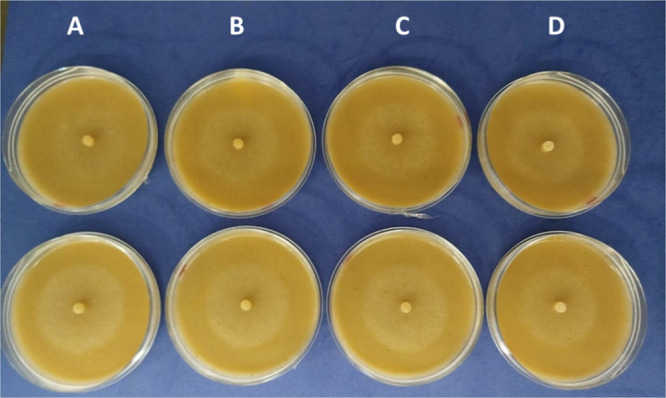
*Phytophthora parasitica* grown in V8 medium with addition of β‐sitosterol 8 days after inoculation. (A) No β‐sitosterol, (B) 1 mg/L β‐sitosterol, (C) 10 mg/L β‐sitosterol, (D) 20 mg/L β‐sitosterol. Treatments shown in replicate confirm no significant differences in mycelial growth.

To evaluate the influence of β‐sitosterol on the metabolites production of *Phytophthora parasitica*, the experiment was conducted with two distinct experimental groups, each containing four treatments. Group 1 (control) consisted of four treatments in absence of *P. parasitica*, at which the plates contained only V8 medium with different concentrations of β‐sitosterol: SV (without sterol addition), SV1 (1 mg/L β‐sitosterol), SV10 (10 mg/L β‐sitosterol) and SV20 (20 mg/L β‐sitosterol). Group 2 included the inoculation of *P. parasitica*, maintaining the same conditions of Group 1, resulting in the treatments SP (V8 medium and *P. parasitica*, without addition of sterol), SP1 (V8 medium, *P. parasitica* and 1 mg/L β‐sitosterol), SP10 (V8 medium, *P. parasitica* and 10 mg/L β‐sitosterol) and SP20 (V8 medium, *P. parasitica* and 20 mg/L β‐sitosterol). The plates containing *P. parasitica* mycelium grown under different conditions were evaluated and diameter of the mycelia was measured. No relevant differences were observed between the treatments (Figure 1; Table 1).

**Table 1:** Diameter of 10 replicates of *Phytophthora parasitica* grown in V8 medium with addition of β‐sitosterol 8 days after inoculation.

β‐sitosterol	1	2	3	4	5	6	7	8	9	10	Media
0	5.2	5.3	5.0	5.4	5.0	4.7	4.8	5.0	5.0	5.1	5.05
1 mg/L	5.1	5.1	5.0	5.2	4.9	5.1	5.1	5.1	4.9	5.4	5.08
10 mg/L	5.2	5.0	5.3	5.3	4.8	5.0	4.8	5.2	5.0	5.3	5.09
20 mg/L	4.9	4.9	4.9	5.0	5.2	5.2	5.0	5.2	5.1	5.3	5.07

*Note:* Data expressed in centimeters (cm).

**Figure 2:**
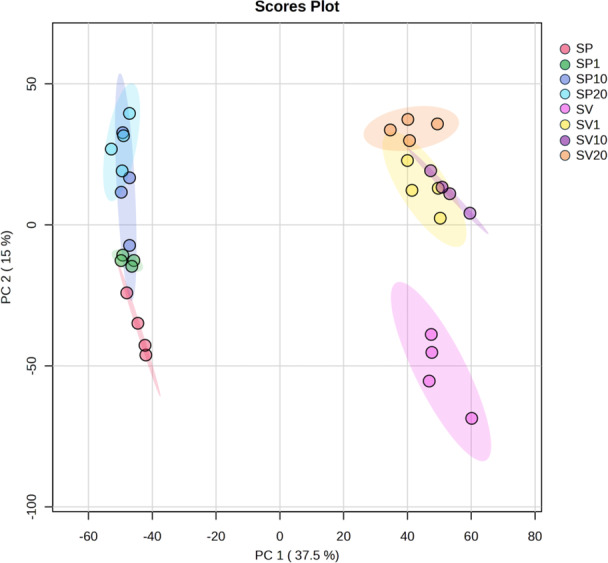
Principal Component Analysis (PCA) score plot generated in MetaboAnalyst, showing the distribution of experimental treatments based on metabolic profiles. The first principal component (PC1) explains 37.5% of the total variance and separates samples primarily by the presence or absence of *Phytophthora parasitica*. The second component (PC2), accounting for 15% of the variance, contributes to further separation among treatments. Distinct clustering patterns are observed: SP, SP1, SP10, and SP20 (left side) represent samples with *P. parasitica*, while SV, SV1, SV10, and SV20 (right side) are pathogen‐free. Each dot represents one biological sample. Gradual shifts along PC1 and PC2 reflect the influence of increasing β‐sitosterol concentrations on the metabolomic profile of each group.

After 10 days of *P. parasitica* growth in SP plates, we arbitrarily chose four plates from each treatment for metabolomics analysis, totalizing 32 samples. After methanol extraction of the metabolites present in the treatments, LC‐HRMS analysis and data processing, we used principal component analysis (PCA) to check for reproducibility and clustering in our samples. PCA revealed that the first component (PC1) accounted for 37.5% of the total variability, while the second component (PC2) explained 15% (Figure 2). This distribution indicates that most of the variation observed in the metabolic data can be attributed to PC1, which strongly discriminates the experimental groups based on the presence of *P. parasitica* and the different concentrations of β‐sitosterol. The graphical visualization of the PCA scores showed that the treatments form distinct clusters, especially when comparing the treatments with and without the pathogen (Figure 2). The influence of β‐sitosterol concentration was also evident, with clear distinctions between the SV group and the SV1, SV10, and SV20 treatments, as well as between SP and SP20 (Figure 2).

We also applied a PERMANOVA analysis, which indicated statistically significant differences between treatments (*p* < 0.05), demonstrating that the metabolic composition of the samples was affected by the experimental factors (Table 2). The coefficient of determination (R²) indicated that a substantial proportion of the variance in the data was explained by the experimental variables, particularly the presence of the pathogen and the availability of β‐sitosterol. The comparisons that did not show statistically significant differences were SP10 versus SP20, SV1 versus SV10, and SV1 versus SV20, suggesting few differential responses between these conditions (Table 2).

**Table 2:** PERMANOVA analysis results generated by MetaboAnalyst comparing pairwise metabolomic profiles among treatments.

Comparison	F.Model	R2	*p*val	*p*.adj	Results
SP versus SP1	20.687	0.77517	0.028	0.04139	Significant
SP versus SP10	21.612	0.7827	0.031	0.04139	Significant
SP versus SP20	79.947	0.93019	0.03	0.04139	Significant
SP versus SV	109.91	0.94824	0.027	0.04139	Significant
SP1 versus SP10	7.43	0.55324	0.032	0.04139	Significant
SP1 versus SP20	60.917	0.91034	0.031	0.04139	Significant
SP1 versus SV1	186.03	0.96876	0.03	0.04139	Significant
SP10 versus SP20	2.0284	0.25266	0.201	0.20844	Not significant
SP10 versus SV10	89.268	0.93702	0.033	0.04139	Significant
SP20 versus SV20	204.25	0.97146	0.027	0.04139	Significant
SV versus SV1	41.435	0.87351	0.034	0.04139	Significant
SV versus SV10	53.562	0.89927	0.041	0.04592	Significant
SV versus SV20	108.61	0.94765	0.023	0.04139	Significant
SV1 versus SV10	1.0761	0.15207	0.348	0.348	Not significant
SV1 verss SV20	4.6438	0.43629	0.075	0.08077	Not significant
SV10 versus SV20	22.627	0.79041	0.029	0.04139	Significant

*Note:* The table shows the pseudo‐F statistic (F.MODEL), which indicates the ratio of variance between groups to variance within groups, the coefficient of determination (R²) representing the proportion of variation explained by group differences, the raw *p*‐value (PVAL) testing the null hypothesis of no difference between groups, the adjusted *p*‐value (P.ADJ) controlling for multiple comparisons using false discovery rate correction, and the significance of each comparison based on the adjusted *p*‐value. Significant differences (*p*.adj < 0.05) indicate statistically distinct metabolic profiles between treatment pairs, while non‐significant results suggest similar profiles.

**Figure 3:**
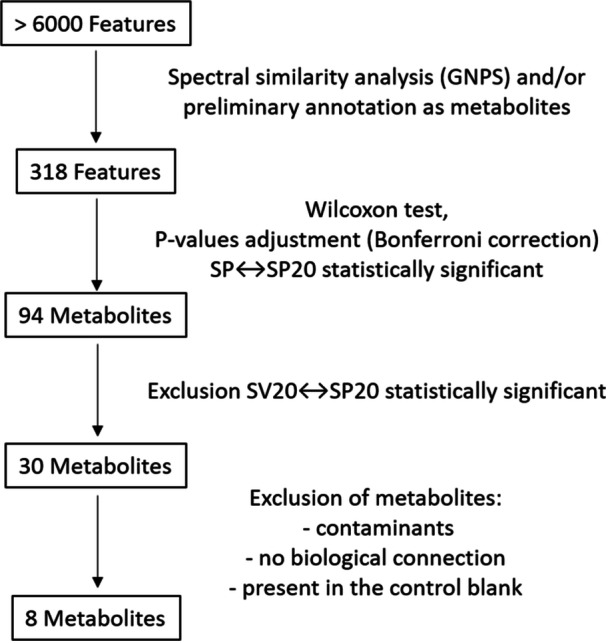
Flowchart summarizing the pipeline selection of metabolites.

Mass spectrometry analysis detected over 6000 features (defined by unique *m/z* and retention time pairs), of which 318 were selected based on spectral similarity and/or preliminary annotation as metabolites. For each of these 318 features, pairwise statistical analysis was performed using the Wilcoxon test, followed by a *P*‐value adjustment using Bonferroni correction, to detect significant variations between treatments. The comparison between SP (V8 medium with *P. parasitica*) and SP20 (V8 medium with *P. parasitica* and 20% β‐sitosterol) was particularly informative, revealing 94 putative metabolites with statistically significant changes. Thereafter, we excluded the metabolites that also significantly varied between SV20 (V8 medium with 20% β‐sitosterol without *P. parasitica*) and SP20. This further step removed metabolites that were not modulated by the concentration of β‐sitosterol, but solely by the oomycete growing. From this list of 30 putative metabolites, signals identified as contaminants, those lacking plausible biological relevance, and metabolites present in control (blank) samples were excluded, resulting in eight more robust metabolites for analysis (Figure 3, Tables 3, 4). The identity of these eight metabolites was then confirmed through spectral analysis using the GNPS database, including only those with a cosine score above 0.8, ensuring high confidence in the annotation (Supporting Information S1), corresponding to level 2 putative identification according to the Metabolomics Standards Initiative (MSI).

**Table 3:** List of metabolites selected after statistical filtering and confirmed by spectral matching in the GNPS library.

Metabolite	Molecular Formula	[M + H]^+^ (*m/z*) exp	Error (ppm)	Global VIPs
Histidine	C_6_H_9_N_3_O_2_	156.0768	−3.2	0.9116666
Aminobenzoic acid	C₇H₇NO₂	138.0550	−3.6	1.0843097
Methylthioadenosine	C₁₁H₁₅N₅O₃S	298.0967	−2.3	1.2594409
Isoleucyl‐Leucine	C₁₂H₂₄N₂O₃	245.1856	−3.7	0.9170975
Cyclo(‐Phe‐Pro)	C₁₄H₁₆N₂O₂	245.1282	−3.3	0.6415743
Sphinganine	C₁₈H₃₉NO₂	302.3055	−1.3	1.0401271
1‐Hexadecanoyl‐sn‐glycerol	C₁₉H₃₈O₄	331.2840	−2.5	1.0550193
Glyceryl monostearate	C₂₁H₄₂O₄	359.3156	−1.5	0.6801834

*Note:* The table shows the metabolite name, molecular formula, experimental *m/z* value for the [M + H]⁺ ion, mass error (ppm) and the VIP values obtained from PLS‐DA analysis in R.

**Table 4:** Paired statistical comparisons, profile variations, and vector similarity for metabolites detected in β‐sitosterol treatments.

row.ID	METABOLITE	row *m/z*	SP x SP20	SV x SP	SV x SV20	SV20 x SP20	SP→SP20	SV→SP	SV→SV20	SV20→SP20	COSINE
150	Histidine	156.0768	**0.0481**	**0.0267**	**0.0246**	0.6857	UP	DOWN	DOWN	EVEN	0.9965
570	Aminobenzoic acid	138.055	**0.0286**	0.6857	**0.0286**	0.8857	DOWN	DOWN	DOWN	EVEN	0.9067
603	Methylthioadenosine	298.0967	**0.0584**	**0.0286**	**0.0211**	NA	DOWN	DOWN	DOWN	EVEN	0.9341
757	Isoleucyl‐leucine	245.1856	**0.0211**	**0.0211**	NA	NA	DOWN	UP	EVEN	EVEN	0.9971
1232	Cyclo(‐Phe‐Pro)	245.1283	**0.0267**	0.6857	0.6857	0.6857	DOWN	UP	EVEN	EVEN	0.8909
3741	Sphinganine	302.3052	**0.0211**	**0.0286**	**0.0584**	NA	DOWN	UP	DOWN	EVEN	0.8505
5552	1‐Hexadecanoyl‐sn‐glycerol	331.284	**0.0286**	**0.0267**	**0.0571**	0.8857	UP	DOWN	UP	EVEN	0.8976
6052	Glyceryl monostearate	359.3153	**0.0265**	**0.0481**	**0.0265**	0.6857	UP	DOWN	UP	EVEN	0.8702

*Note:* The table presents the results of the paired Wilcoxon test for all detected metabolites, considering four specific comparisons: SP versus SP20, SV versus SP, SV versus SV20, and SV20 versus SP20. The table includes the ID of each metabolite as identified in MZmine, the adjusted *p*‐values resulting from the Wilcoxon test, and highlights in bold those comparisons that showed statistically significant differences (*p* < 0.05). It also lists the name of the putative or confirmed metabolite (column “METABOLITE”) and its corresponding mass‐to‐charge ratio (column “row *m/z*”). The columns “SP → SP20,” “SV → SP,” “SV → SV20,” and “SV20 → SP20” qualitatively describe the direction of change in relative abundance between treatments. The “COSINE” column refers to the vector similarity between the abundance profiles oftreatments SP and SV across the concentrations, as calculated by GNPS2.

**Figure 4:**
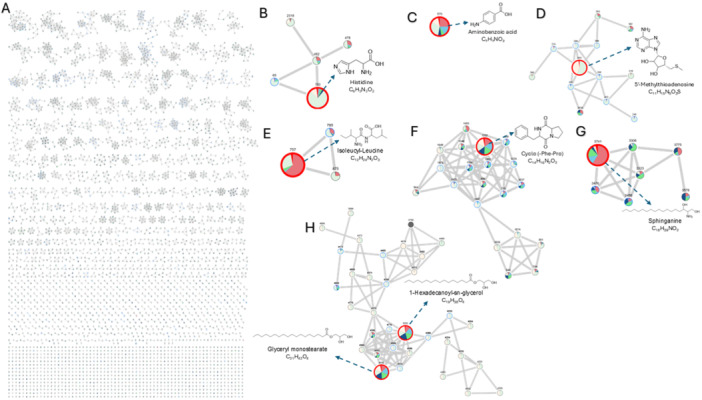
Global molecular network and highlighting of eight identified metabolites. A. Molecular network constructed from fragmentation spectra, showing structural relationships among the detected metabolites. Eight selected metabolites are highlighted in the network: B ‐ histidine; C ‐ aminobenzoic acid (singleton); D ‐ 5'‐methylthioadenosine; E ‐ isoleucyl‐leucine; F ‐ cyclo(‐Phe‐Pro); G ‐ sphinganine; H ‐ 1‐hexadecanoyl‐sn‐glycerol and glyceryl monostearate.

The constructed molecular network from fragmentation spectra highlighted the structural relationships among the detected metabolites. Of the eight metabolites selected, seven were allocated into clusters, whereas the ion *m/z* [M + H]^+^ 138.0550 was observed as a single node, indicating insufficient spectral similarity for clustering. Two metabolites (*m/z* [M + H]^+^331.2840 and *m/z* [M + H]^+^ 359.3153) were grouped within the same cluster, suggesting structural proximity. The remaining metabolites were distributed across different clusters, each connected to structurally related compounds, indicating that they belong to distinct chemical families (Figure 4).

**Figure 5:**
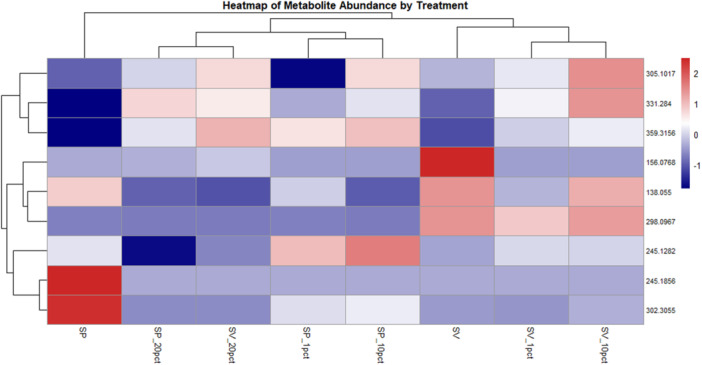
Metabolomic heatmap of *Phytophthora parasitica* response to β‐sitosterol supplementation. Heatmap representing the relative abundance (z‐score scaled) of selected metabolites across eight experimental treatments. Rows correspond to individual metabolites, and columns to treatment conditions: SP (medium with *Phytophthora parasitica*, no sterol), SP1, SP10, SP20 (with 1 mg/L, 10 mg/L, 20 mg/L β‐sitosterol, respectively), and SV (medium without *Phytophthora*, no sterol), SV1, SV10, SV20 (with 1 mg/L, 10 mg/L, 20 mg/L β‐sitosterol, respectively). Red indicates higher relative abundance, and blue indicates lower relative abundance compared to the mean abundance of each metabolite across treatments. Both metabolites and treatments were clustered using hierarchical clustering (Euclidean distance, complete linkage) to reveal patterns of similarity. The heatmap suggests sterol concentration and presence of *Phytophthora* distinctly influence the metabolomic profile.

The evaluation of the profiles of these eight metabolites revealed different response patterns to the experimental conditions. Two metabolites at *m/z*[M + H]^+^ 331.2840 (Row. ID 5552), and *m/z* [M + H]^+^ 359.3153 (Row. ID 6052) showed a pattern compatible with metabolic activation of the pathogen specifically in the presence of β‐sitosterol. Other compounds, such as the metabolite at *m/z* [M + H]^+^ 156.0768 (Row. ID 150), showed more complex regulation, indicating that its modulation may result from the combined interaction between the pathogen, β‐sitosterol, and the culture medium. In addition, metabolite at *m/z*[M + H]^+^ 245.1856 (Row. ID 757) was detected only in the treatment with *P. parasitica* in the absence of β‐sitosterol, while the compound at *m/z*[M + H]^+^ 298.0967 (Row. ID 603) showed a progressive reduction when both the pathogen and β‐sitosterol were present, suggesting joint suppression. Metabolite ion at *m/z* [M + H]^+^ 302.3055 (Row. ID 3741) exhibited a profile indicative of inhibition by the presence of sterol, with or without the pathogen. A particularly interesting case was metabolite ion at *m/z* [M + H]^+^ 245.1282 (Row. ID 1232), whose cross‐regulation shows production stimulated by the presence of the pathogen and suppression induced by β‐sitosterol, without influence from the medium (Table 4). These distinct modulation patterns are visually represented in the hierarchical clustering heatmap (Figure 5), which illustrates the relative abundance profiles of metabolites across treatments and reveals treatment‐driven groupings and co‐regulation patterns of metabolites.

**Figure 6:**
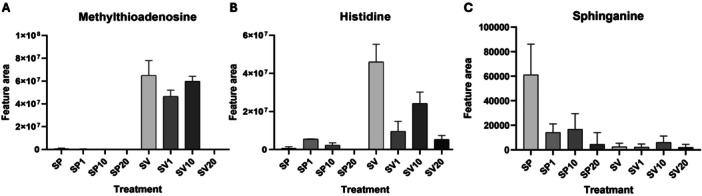
Differential accumulation of selected metabolites in response to β‐sitosterol in the presence or absence of *Phytophthora parasitica*. Bar plots showing the mean MS intensity of three selected metabolites across eight experimental treatments combining *Phytophthora parasitica* (SP) or pathogen‐free control (SV) with increasing concentrations of β‐sitosterol 1 mg/L, 10 mg/L and 20 mg/L, corresponding to SP, SP1, SP10, SP20 and SV, SV1, SV10, SV20, respectively. (A) 5′‐methylthioadenosine (MTA), (B) L‐histidine and (C) sphinganine. Bars represent mean ± SE (*n* = 4).

Three metabolites were selected for detailed analysis based on their biological relevance, response patterns (Table 4), and their VIP (Variable Importance in Projection) values obtained from PLS‐DA (Partial Least Squares Discriminant Analysis) (Supporting Information S2), considering a VIP threshold of 0.9 (Table 3): 5′‐methylthioadenosine, L‐histidine, and sphinganine. These modulation profiles can also be observed in an integrated manner in Figure 6, which depicts the response trajectories of metabolites across the different treatments.

## 4 Discussion

The great majority of metabolomics studies regarding *Phytophthora* are interested in metabolic profiles of plants challenged by these oomycete [23, 24, 25, 26]. Recently, aspects of metabolic *Phytophthora* responses to environment have been evaluated, as are the cases of the synergistic effects of combined treatment of the chemical defensives dimethomorph (DIM) and pyrimethanil (PYM) against *P. capsici* [27] and the lipidomics of *P. sojae* after the effects of the treatment of oxathiapiprolin, an oxysterol‐binding protein [28]. Strikingly, Maximo and collaborators [29] evaluated the metabolic profile of *P. parasitica* under the influence of *Citrus sunki* (a highly susceptible host) and *Poncirus trifoliata* (a resistant genotype) extracts, identifying an amino acid, two dipeptides and other unknown metabolites, whose production was modulated by the root extracts. Our study provides a novelty to this scenario, exploring the metabolic reprogramming of *Phytophthora parasitica* upon different concentrations of β‐sitosterol, knowing the importance of sterols in the extracellular environment of sterol‐auxotrophic *Peronosporales* oomycetes.

Multivariate analysis revealed that both the presence of *P. parasitica* and the different concentrations of β‐sitosterol significantly affected the metabolic profiles of the samples. Principal Component Analysis (PCA) showed that the majority of variance (PC1 = 37.5%) was explained by these two factors, highlighting their predominant influence. The clear separation between treatment groups reinforces the idea that β‐sitosterol acts as a relevant physiological modulator for the pathogen, supporting previous findings that plant sterols directly affect growth, sporulation, and cellular signaling in sterol‐auxotrophic oomycetes [30, 31, 32]. The statistical significance observed in the PERMANOVA analysis (*p* < 0.05), along with the substantial R² values, confirms that the experimental factors explain a considerable portion of the observed variability. Moreover, the lack of difference between some group comparisons, such as SV1 versus SV20, suggests a dose‐dependent behavior up to a threshold, followed by response stabilization. This may reflect saturation of β‐sitosterol uptake or signaling mechanisms, as previously reported in *P. sojae* [31] and *P. sojae* [33]. The filtering of features detected by LC‐HRMS allowed us to isolate the pathogen's response to β‐sitosterol, annotating 30 putative metabolites potentially modulated by this interaction. Spectral validation resulted in 8 confidently annotated compounds, many of which showed distinct modulation patterns. This indicates that β‐sitosterol not only directly influences metabolism but also interacts with pathways regulated by the presence of the pathogen. This is consistent with the known role of elicitins and sterol‐sensing domain (SSD)‐containing proteins in mediating sterol perception and response in oomycetes [34, 35].

The detection of metabolites jointly suppressed by both β‐sitosterol and the pathogen points to potential mechanisms of metabolic repression or redirection of biosynthetic pathways. This may reflect an adaptation to the altered lipid equilibrium established in the presence of exogenous sterols. These findings reinforce that β‐sitosterol plays a multifunctional role in *P. parasitica* physiology, influencing not only membrane composition but also broader metabolic regulatory processes [36, 37]. Overall, our results support the notion that the abundance or depletion of β‐sitosterol in the environment serves as a key ecological signal for *P. parasitica*, capable of inducing extensive metabolic reprogramming. This has direct implications for understanding pathogenicity mechanisms and may inform management strategies that exploit the sterol dependence of these pathogens. Intriguingly, 1‐hexadecanoyl‐sn‐glycerol and glyceryl monostearate were found as UP in SV x SV20 comparison (Table 4). Although speculative, this increasing can be caused by a non‐enzymatic reaction between β‐sitosterol and components of the milieu [38]. Nevertheless, the fact that these metabolites are DOWN in SV x SP (Table 4), made us consider them in our final data, as we infer that the oomycete likely consumed them from the culture medium.

Due to their biological relevance and significant variations in response to the presence of the oomycete *P. parasitica* and the phytosterol β‐sitosterol, three metabolites are discussion with more depth. The choice of these compounds is supported by evidence indicating their roles in virulence, metabolic regulation, and signaling during the pathogen‐host interaction, especially in the context of sterol recognition.

MTA (5'‐methylthioadenosine) is a sulfur‐containing nucleoside derived from S‐adenosylmethionine via the polyamine pathway and also participates in the methionine salvage pathway. Studies have shown that this metabolite regulates key cellular processes such as gene expression and proliferation [39]. In *P. infestans*, folate‐biopterin transporters (FBTs) comprise an unusually large family, whose members are differentially expressed throughout oomycete development [40]. FBTs take up cofactors for methionine and purine synthesis, indicating that the connected folate cycles, methionine cycles and methionine salvage pathway cycles are important for *Phytophthora* development. In many pathogens, MTA has been associated with virulence modulation. For instance, Bourgeois and collaborators [41] demonstrated that in *Salmonella enterica* serovar Typhimurium, MTA suppresses bacterial motility and host cell invasion by inhibiting the flagellar and SPI‐1 systems, resulting in reduced in vivo virulence. These findings suggest that MTA acts as a negative regulator of virulence in *Salmonella*, by silencing pathogenic gene networks. In our data, a marked low level of MTA was observed in treatments containing the pathogen (SP) suggesting that the oomycete may be degrading or rapidly utilizing this metabolite under sterol starvation. It reflects the activation of methionine salvage and polyamine biosynthesis pathways by the pathogen, leading to rapid consumption of the compound. Future studies should directly explore the relationship between MTA levels and the expression of virulence genes in *P. parasitica*.

L‐histidine is an essential amino acid with multiple physiological functions, acting as a precursor of histamine, a regulator of cellular redox status, and a metal‐chelating agent for iron, zinc, and copper [42, 43]. In fungi and oomycetes, histidine regulation is directly related to nutrient homeostasis—especially metals such as iron, copper, and zinc—and to adaptation to adverse environmental conditions, including overcoming host defenses [43]. In the present study, the levels of this metabolite showed an oscillating pattern, with a notable high abundance in the SV treatment (pure V8 medium, without pathogen). The high intensity detected in SV can be explained by the composition of the V8 medium, which is rich in vegetable extracts providing free amino acids, including histidine. The addition of β‐sitosterol produced non‐linear effects on histidine levels. In the absence of sterol (SP treatment), histidine levels are much lower than in SV treatment suggesting its rapid consumption or redirection to metabolic pathways crucial for oomycete adaptation. Besides acting as a redox buffer, histidine plays important roles in the pathogen's response to reactive oxygen species (ROS), suggesting a relevant antioxidant function during oxidative stress imposed by host defenses [44]. In *P. cactorum* and *P. heveae*, histidine can even be used as a nitrogen source in minimal media [45], reinforcing its importance under nutrient‐limiting conditions. This metabolic versatility may also support overcoming host defense barriers by sustaining processes such as siderophore synthesis, effector protein secretion, and apoplastic pH regulation, which depend on specific amino acids and metal availability [46, 47]. Studies with *P. infestans* demonstrate that genes related to amino acid degradation, such as histidine ammonia‐lyase (HAL), are expressed during infection, suggesting the use of histidine as a nitrogen and carbon source in nutrient‐rich environments such as plant tissues [6]. Moreover, histidine may act as a redox buffer and contribute to nucleotide and coenzyme synthesis, critical processes during stress and colonization.

Sphinganine (also known as dihydrosphingosine) is a long‐chain sphingoid base essential for sphingolipid biosynthesis. It is produced via the condensation of serine with a long‐chain fatty acid by the serine palmitoyltransferase (SPT) complex in the endoplasmic reticulum, and is rapidly converted into ceramide, serving as a structural precursor for complex sphingolipids [48, 49]. In fungi and oomycetes, sphinganine gives rise to essential membrane sphingolipids. In yeasts and filamentous fungi, for instance, ceramides and glycosphingolipids commonly contain sphingosine (d18:1) or its saturated form, sphinganine (d18:0), as the long‐chain base [50]. *Phytophthora parasitica* and *P. capsici* synthesize ceramide‐type phosphoinositide sphingolipids, with sphinganine (C16‐dihydrosphingosine) as the long‐chain base linked to saturated or monounsaturated fatty acids [51, 52]. In *P. parasitica*, sphingolipids include C16 sphinganine acylated with long‐chain fatty acids (16:0, 20:0, 22:0, etc.). These lipids differ from those in fungi, which are typically dominated by glycosphingolipids such as GIPC or GlcCer, while in *Phytophthora*, most molecules are phosphoinositol ceramides lacking sugar moieties [50, 51]. Nonetheless, the presence of sphinganine as a key component suggests conserved physiological roles, including membrane microdomain formation, regulation of endocytosis, and promotion of cell growth, such as enrichment of sphingolipids at fungal bud necks [50]. Environmental release of sphingolipids may also influence host–pathogen interactions by signaling physiological changes or disrupting host membranes [50]. As mentioned above, *P. parasitica* cannot synthesize sterols, acquiring it from the environment or host [33, 35]. Sterols and sphingolipids strongly interact in membrane lipid rafts, where they are densely packed and participate in signaling and protein adhesion [50]. In the experimental context involving β‐sitosterol (SP20), its addition led to a reduction in free sphinganine levels. One hypothesis is that β‐sitosterol incorporation into membranes reduces the demand for *de novo* sphingolipid synthesis or alters lipid raft dynamics, negatively regulating sphinganine biosynthesis. Involvement of oxysterol‐binding proteins (OSBPs), which mediate sterol and sphingolipid exchange between organelles, is also possible. In *P. sojae*, the OSBP inhibitor oxathiapiprolin caused accumulation of free sphingosine and sitosterol esters, indicating that normal sphingolipid transport depends on OSBP function [28]. Thus, in the presence of free β‐sitosterol, *P. parasitica* may downregulate the sphingolipid pathway to prevent membrane lipid excess, reducing the sphinganine pool. Interestingly, sphingolipids have been characterized as putative oomycete elicitors of plant defense [52, 53]. Actually, the interaction between sterol and sphingolipids can be used as targets of plant protection against pathogens, through the disruption of lipid rafts or using sphingolipids as elicitors of plant defense, or as sequestering agents of sterol leading to oomycete starvation [54, 55, 56]. It is tempting to suggest that the higher amount of sphinganine in a low sterol milieu may mimic a sterol‐deprived interface during pathogen infection increasing sphingolipid content in the oomycete membrane. In summary, exogenous sterol availability may indirectly modulate sphingolipid production through regulatory mechanisms yet to be fully elucidated in oomycetes [28, 35].

Our findings reveal that β‐sitosterol exerts an impact on the metabolic landscape of *P. parasitica*, promoting a concentration‐dependent shift in key biosynthetic and signaling pathways. The differential behavior of metabolites such as 5′‐methylthioadenosine, L‐histidine and sphinganine underscores a coordinated metabolic response rather than isolated alterations. This response likely reflects the pathogen's capacity to perceive and integrate exogenous sterols as cues for adjusting its physiology. Rather than functioning only as structural components, sterols such as β‐sitosterol appear to act as modulators of oomycete metabolism, influencing processes central to survival and pathogenicity.

Our data can add new possibilities to practical applications in disease management. For instance, the possible consumption of MTA and histidine by the pathogen (Figure 6), indicates that modulation of methionine and histidine metabolism can be a good target for plant pathogen control [57]. As mentioned above, sphingolipids as sphinganine are known as potential elicitors of plant defense [53]. The use of this compound as a priming defense molecule may be an attractive strategy to halt *P. parasitca* infection. By employing a metabolomics‐based approach, this study contributes to a deeper understanding of sterol‐pathogen interactions and opens new avenues for exploring metabolite‐mediated strategies for disease management and interference with virulence signaling. These results highlight the importance of the studied metabolites as mediators of the oomycete's adaptive responses to the environment, paving the way for future investigations into the molecular mechanisms underlying virulence modulation via sterol signaling.

## Author Contributions

**Pamela Ponce Martins:** conceptualization, methodology, formal analysis, investigation, writing – original draft, writing – review and editing. **Evandro Silva:** conceptualization, formal analysis, writing – original draft, writing – review and editing. **Marcus Vinicius Fernandes Prior:** methodology, writing – review and editing. **João Marcos Martins Ferreira:** methodology, writing – review and editing. **Marina Erê Pimenta Santos:** methodology, writing – review and editing. **Flavia Rodrigues Alves Patrício:** methodology, writing – review and editing. **Taicia Pacheco Fill:** conceptualization, writing – review and editing, supervision, resources. **Jorge Maurício Costa Mondego:** conceptualization, writing – original draft, writing – review and editing, supervision, resources, project administration.

## Conflicts of Interest

The authors declare no conflicts of interest.

## Supporting information

749Supporting Information 1.

749Supporting Information 2.

## Data Availability

The data that support the findings of this study are available from the corresponding author upon reasonable request.

## References

[1] F. Panabières , S. Gul , G. Ali , et al., “ *Phytophthora nicotianae* Diseases Worldwide: New Knowledge of a Long‐Recognised Pathogen,” Phytopathologia Mediterranea 55 (2016): 20–40, 10.14601/Phytopathol_Mediterr-16423.

[2] M. P. Caixeta , W. M. C. Nunes , dos , A. F. Santos , D. J. Tessmann , and J. B. Vida , “Espécies de *Phytophthora* associadas à gomose em pomares de citros no Estado do Paraná,” Brasil Summa Phytopathologica 39, no. 4 (2013): 242–247, 10.1590/S0100-54052013000400002.

[3] E. Feichtenberger , “Control of *Phytophthora* Gummosis of Citrus With Systemic Fungicides in Brazil,” EPPO Bulletin 20, no. 1 (1990): 139–148, 10.1111/j.1365-2338.1990.tb01191.x.

[4] R. J. D. Dalio , H. J. Máximo , T. S. Oliveira , et al., “Molecular Basis of *Citrus sunki* Susceptibility and *Poncirus trifoliata* Resistance Upon *Phytophthora parasitica* Attack,” Molecular Plant‐Microbe Interactions® 31, no. 3 (2018): 386–398, 10.1094/MPMI-05-17-0112-FI. 29125028

[5] M. F. Vidal and L. Citricultura Caderno Setorial ETENE 2024; 9, 328, Available at: https://www.bnb.gov.br/revista/cse/article/view/2621.

[6] H. S. Judelson and F. A. Blanco , “The Spores of *Phytophthora*: Weapons of the Plant Destroyer,” Nature Reviews Microbiology 3, no. 1 (2005): 47–58, 10.1038/nrmicro1064. 15608699

[7] M. Kasteel , T. P. Rajamuthu , J. Sprakel , T. Ketelaar , and F. Govers , “ *Phytophthora* Zoospores Display Klinokinetic Behaviour in Response to a Chemoattractant,” PLoS Pathogens 20, no. 9 (2024): e1012577, 10.1371/journal.ppat.1012577. 39348406 PMC11554144

[8] J. Chepsergon , T. E. Motaung , D. Bellieny‐Rabelo , and L. N. Moleleki , “Organize, Don't Agonize: Strategic Success of *Phytophthora* Species,” Microorganisms 8, no. 6 (2020): 917, 10.3390/microorganisms8060917. 32560346 PMC7355776

[9] J. W. Hendrix and S. M. Guttman , “Sterol or Calcium Requirement by *Phytophthora parasitica* var. Nicotianae for Growth on Nitrate Nitrogen,” Mycologia 62, no. 1 (1970): 195–198. 5441001

[10] P. Dahlin and A. C. Ruthes , “Loss of Sterol Biosynthesis in Economically Important Plant Pests and Pathogens: A Review of a Potential Target for Pest Control,” Biomolecules 14, no. 11 (2024): 1435, 10.3390/biom14111435. 39595611 PMC11591786

[11] M. Fabris , M. Matthijs , S. Carbonelle , et al., “Tracking the Sterol Biosynthesis Pathway of the Diatom *Phaeodactylum tricornutum* ,” New Phytologist 204, no. 3 (2014): 521–535, 10.1111/nph.12917. 24996048

[12] D. Gottlieb , “Differences in the Sterol Synthesizing Pathways of Sterol Producing and Non‐Sterol Producing Fungi,” Phytopathology 68 (1978): 1168–1169.

[13] C. G. Elliott , “Sterols and the Production of Oospores by *Phytophthora cactorum* ,” Journal of General Microbiology 72 (1972): 321–327.

[14] C. G. Elliott , M. R. Hendrie , and B. A. Knights , “The Sterol Requirement of *Phytophthora cactorum* ,” Journal of General Microbiology 42, no. 3 (1966): 425–435. 5915375 10.1099/00221287-42-3-425

[15] M. Kopischke , L. Westphal , K. Schneeberger , et al., “Impaired Sterol Ester Synthesis Alters the Response of *Arabidopsis thaliana* to *Phytophthora infestans* ,” Plant Journal 73, no. 3 (2013): 456–468, 10.1111/tpj.12046. 23072470

[16] K. Stenzel and J. P. Vors Sterol Biosynthesis Inhibitors. In: Jeschke P., Witschel M., Krämer W, Schirmer U. editors. Modern Crop Protection Compounds. 3rd ed. Vol. 2. Wiley‐VCH; Weinheim, Germany: 2019. 797–844.

[17] C. Der , P. E. Courty , G. Recorbet , D. Wipf , F. Simon‐Plas , and P. Gerbeau‐Pissot , “Sterols, Pleiotropic Players in Plant‐Microbe Interactions,” Trends in Plant Science 29, no. 5 (2024): 524–534, 10.1016/j.tplants.2024.03.002. 38565452

[18] S. Romero and M. E. Gallegly , “Oogonium Germination in *Phytophthora infestans* ,” Phytopathology 53, no. 8 (1963): 899–903.

[19] E. Silva , R. Dantas , J. C. Barbosa , R. G. S. Berlinck , and T. Fill , “Metabolomics Approach to Understand Molecular Mechanisms Involved in Fungal Pathogen‐Citrus Pathosystems,” Molecular Omics 20, no. 3 (2024): 154–168, 10.1039/d3mo00182b. 38273771

[20] M. C. Chambers , B. Maclean , R. Burke , et al., “A Cross‐Platform Toolkit for Mass Spectrometry and Proteomics,” Nature Biotechnology 30, no. 10 (2012): 918–920, 10.1038/nbt.2377. PMC3471674 23051804

[21] T. Pluskal , S. Castillo , A. Villar‐Briones , and M. Orešič , “MZmine 2: Modular Framework for Processing, Visualizing, and Analyzing Mass Spectrometry‐Based Molecular Profile Data,” BMC Bioinformatics 11 (2010): 395, 10.1186/1471-2105-11-395. 20650010 PMC2918584

[22] L. F. Nothias , D. Petras , R. Schmid , et al., “Feature‐Based Molecular Networking in the GNPS Analysis Environment,” Nature Methods 17, no. 9 (2020): 905–908, 10.1038/s41592-020-0933-6. 32839597 PMC7885687

[23] P. Kruaweangmol , K. Ekchaweng , S. Morakul , N. Phaonakrop , S. Roytrakul , and P. Tunsagool , “Metabolomic and Proteomic Changes in Leaves of Rubber Seedlings Infected by *Phytophthora palmivora* ,” Tree Physiology 45, no. 2 (2025): tpaf010, 10.1093/treephys/tpaf010. 39869784

[24] G. Lei , K. H. Zhou , X. J. Chen , et al., “Transcriptome and Metabolome Analyses Revealed the Response Mechanism of Pepper Roots to *Phytophthora capsici* Infection,” BMC Genomics 24, no. 1 (2023): 626, 10.1186/s12864-023-09713-7. 37864214 PMC10589972

[25] D. Neves , A. Figueiredo , M. Maia , E. Laczko , M. S. Pais , and A. Cravador , “A Metabolome Analysis and the Immunity of *Phlomis purpurea* Against *Phytophthora cinnamomi* ,” Plants (Basel, Switzerland) 12, no. 10 (2023): 1929, 10.3390/plants12101929. 37653845 PMC10223286

[26] I. Saiz‐Fernández , B. Đorđević , P. Kerchev , et al., “Differences in the Proteomic and Metabolomic Response of *Quercus suber* and *Quercus variabilis* During the Early Stages of *Phytophthora cinnamomi* Infection,” Frontiers in Microbiology 13 (2022): 894533, 10.3389/fmicb.2022.894533. 35770156 PMC9234522

[27] Y. Zhang , L. Zhou , C. Wang , and S. Liu , “Synergistic Antifungal Effect and Potential Mechanism of Dimethomorph Combined With Pyrimethanil Against *Phytophthora capsici* ,” Food Chemistry 457 (2024): 140158, 10.1016/j.foodchem.2024.140158. 38936133

[28] X. Liu , C. Li , Y. Chen , Z. Xue , J. Miao , and X. Liu , “Untargeted Lipidomics Reveals Lipid Metabolism Disorders Induced by Oxathiapiprolin in *Phytophthora sojae* ,” Pest Management Science 79, no. 4 (2023): 1593–1603, 10.1002/ps.7334. 36562252

[29] H. J. Maximo , F. D. S. Araújo , C. C. Pagotto , et al., “Influence of *Citrus sunki* and *Poncirus trifoliata* Root Extracts on Metabolome of *Phytophthora parasitica* ,” Metabolites 14, no. 4 (2024): 206, 10.3390/metabo14040206. 38668334 PMC11052222

[30] W. D. Dotson , S. R. Tove , and L. W. Parks , “Biochemical Modifications and Transcriptional Alterations Attendant to Sterol Feeding in *Phytophthora parasitica* ,” Lipids 35, no. 3 (2000): 243–247, 10.1007/s11745-000-0519-9. 10783000

[31] Y. Pei , P. Ji , J. Miao , et al., “A Receptor Kinase Senses Sterol by Coupling With Elicitins in Auxotrophic *Phytophthora* ,” Proceedings of the National Academy of Sciences 121, no. 45 (2024): e2408186121, 10.1073/pnas.2408186121. PMC11551405 39475635

[32] W. Wang , F. Zhang , S. Zhang , et al., “ *Phytophthora capsici* Sterol Reductase PcDHCR7 has a Role in Mycelium Development and Pathogenicity,” Open Biology 12, no. 4 (2022a): 210282, 10.1098/rsob.210282. 35382565 PMC8984297

[33] W. Wang , T. Cui , F. Zhang , Z. Xue , B. Zhang , and X. Liu , “Functional Analysis of the C‐5 Sterol Desaturase PcErg3 in the Sterol Auxotrophic Oomycete Pathogen *Phytophthora capsici* ,” Frontiers in Microbiology 13 (2022b): 811132, 10.3389/fmicb.2022.811132. 35651492 PMC9151008

[34] H. Osman , S. Vauthrin , V. Mikes , et al., “Mediation of Elicitin Activity on Tobacco Is Assumed by Elicitin‐Sterol Complexes,” Molecular Biology of the Cell 12, no. 9 (2001): 2825–2834, 10.1091/mbc.12.9.2825. 11553720 PMC59716

[35] W. Wang , X. Liu , and F. Govers , “The Mysterious Route of Sterols in Oomycetes,” PLoS Pathogens 17, no. 6 (2021): e1009591, 10.1371/journal.ppat.1009591. 34138975 PMC8211186

[36] E. Gaulin , A. Bottin , and B. Dumas , “Sterol Biosynthesis in Oomycete Pathogens,” Plant Signaling & Behavior 5, no. 3 (2010): 258–260, 10.4161/psb.5.3.10551. 20023385 PMC2881271

[37] M. Thines , “Oomycetes,” Current Biology 28, no. 15 (2018): R812–R813, 10.1016/j.cub.2018.05.062. 30086308

[38] M. A. Keller , G. Piedrafita , and M. Ralser , “The Widespread Role of Non‐Enzymatic Reactions in Cellular Metabolism,” Current Opinion in Biotechnology 34 (2015): 153–161, 10.1016/jcopbio.2014.10.020. 25617827 PMC4728180

[39] A. Avila , E. R. García‐Trevijano , S. C. Lu , F. J. Corrales , and J. M. Mato , “Methylthioadenosine,” International Journal of Biochemistry & Cell Biology 36, no. 11 (2004): 2125–2130, 10.1016/j.biocel.2003.11.016. 15313459

[40] A. M. V. Ah‐Fong , K. S. Kim , and H. S. Judelson , “RNA‐seq of Life Stages of the Oomycete *Phytophthora infestans* Reveals Dynamic Changes in Metabolic, Signal Transduction, and Pathogenesis Genes and a Major Role for Calcium Signaling in Development,” BMC Genomics 18, no. 1 (2017): 198, 10.1186/s12864-017-3585-x. 28228125 PMC5322657

[41] J. S. Bourgeois , D. Zhou , T. L. M. Thurston , J. J. Gilchrist , and D. C. Ko , “Methylthioadenosine Suppresses *Salmonella* Virulence,” Infection and Immunity 86, no. 9 (2018): e00429‐18, 10.1128/IAI.00429-18. PMC6105896 29866910

[42] T. N. Andrade , B. F. D. Ramirez , G. A. Camargo , and M. T. B. Pacheco , “Revisão das rotas metabólicas dos aminoácidos essenciais: individuais, sinérgicos, BCAAs e sulfurados,” Observatorio de La Economía Latinoamericana 22, no. 8 (2013): e6327, 10.55905/oelv22n8-161.

[43] A. M. Dietl , J. Amich , S. Leal , et al., “Histidine Biosynthesis Plays a Crucial Role in Metal Homeostasis and Virulence of *Aspergillus fumigatus* ,” Virulence 7, no. 4 (2016): 465–476, 10.1080/21505594.2016.1146848. 26854126 PMC4871644

[44] R. Nostadt , M. Hilbert , S. Nizam , et al., “A Secreted Fungal Histidine‐ and Alanine‐Rich Protein Regulates Metal Ion Homeostasis and Oxidative Stress,” New Phytologist 227, no. 4 (2020): 1174–1188, 10.1111/nph.16606. 32285459

[45] J. A. Leal , M. E. Gallegly , and V. G. Lilly , “The Value of 21 Amino Acids as Nitrogen Sources for *Phytophthora cactorum* and *P. heveae* ,” Canadian Journal of Microbiology 17, no. 10 (1971): 1319–1325, 10.1139/m71-211. 5131756

[46] H. Haas , M. Eisendle , and B. G. Turgeon , “Siderophores in Fungal Physiology and Virulence,” Annual Review of Phytopathology 46 (2008): 149–187, 10.1146/annurev.phyto.45.062806.094338. 18680426

[47] S. L. Newman and A. G. Smulian , “Iron Uptake and Virulence in *Histoplasma capsulatum* ,” Current Opinion in Microbiology 16, no. 6 (2013): 700–707, 10.1016/j.mib.2013.09.001. 24094809

[48] K. Hirschberg , J. Rodger , and A. H. Futerman , “The Long‐Chain Sphingoid Base of Sphingolipids Is Acylated at the Cytosolic Surface of the Endoplasmic Reticulum in Rat Liver,” Biochemical Journal 290, no. 3 (1993): 751–757, 10.1042/bj2900751. 8457204 PMC1132344

[49] A. H. Merrill , E. Wang , and R. E. Mullins , “Kinetics of Long‐Chain (Sphingoid) Base Biosynthesis in Intact LM Cells: Effects of Varying the Extracellular Concentrations of Serine and Fatty Acid Precursors of This Pathway,” Biochemistry 27, no. 1 (1988): 340–345, 10.1021/bi00401a051. 3126810

[50] X. M. Zhu , L. Li , J. D. Bao , et al., “The Biological Functions of Sphingolipids in Plant Pathogenic Fungi,” PLoS Pathogens 19, no. 11 (2023): e1011733, 10.1371/journal.ppat.1011733. 37943805 PMC10635517

[51] M. Bruneteau , F. Fournol , C. Gandon , M. Becchi , and V. Pivot , “Isolation and Characterization of Inositol Sphingophospholipids From *Phytophthora parasitica* Dastur,” Lipids 32, no. 4 (1997): 359–362, 10.1007/s11745-997-0045-9. 9113622

[52] H. Kato , K. Nemoto , M. Shimizu , et al., “Recognition of Pathogen‐Derived Sphingolipids in *Arabidopsis* ,” Science 376, no. 6595 (2022): 857–860, 10.1126/science.abn0650. 35587979

[53] O. Lhomme , M. Bruneteau , C. E. Costello , et al., “Structural Investigations and Biological Activity of Inositol Sphingophospholipids From *Phytophthora capsici* ,” European Journal of Biochemistry 191, no. 1 (1990): 203–209, 10.1111/j.1432-1033.1990.tb19111.x. 2379501

[54] M. S. Monjil , H. Kato , S. Ota , et al., “Two Structurally Different Oomycete Lipophilic Microbe‐Associated Molecular Patterns Induce Distinctive Plant Immune Responses,” Plant Physiology 196, no. 1 (2024): 479–494, 10.1093/plphys/kiae255. 38828881 PMC11376384

[55] U. Ali , H. Li , X. Wang , and L. Guo , “Emerging Roles of Sphingolipid Signaling in Plant Response to Biotic and Abiotic Stresses,” Molecular Plant 11, no. 11 (2018): 1328–1343, 10.1016/j.molp.2018.10.001. 30336328

[56] E. Kuźniak and E. Gajewska , “Lipids and Lipid‐Mediated Signaling in Plant‐Pathogen Interactions,” International Journal of Molecular Sciences 25, no. 13 (2024): 7255. 39000361 10.3390/ijms25137255 PMC11241471

[57] J. Moormann , B. Heinemann , and T. M. Hildebrandt , “News About Amino Acid Metabolism in Plant‐Microbe Interactions,” Trends in Biochemical Sciences 47, no. 10 (2022): 839–850, 10.1016/j.tibs.2022.07.001. 35927139

