# Supplementary material for: Metabolomic Analysis of Phytophthora parasitica Growth in the Presence of β‐sitosterol Indicates Adaptive Mechanisms Modulated by Sterols
Source: J Basic Microbiol. 2026 Feb 4;66(2):e70149. doi: 10.1002/jobm.70149 (PMC12873457; doi:10.1002/jobm.70149)

**Supporting Information 1**

Mirror matches of the nine metabolites selected after filtering based on cosine score and biological relevance. The spectral comparisons between experimental and reference spectra illustrate the confidence in metabolite annotation and support the interpretation of their biological modulation across treatments.


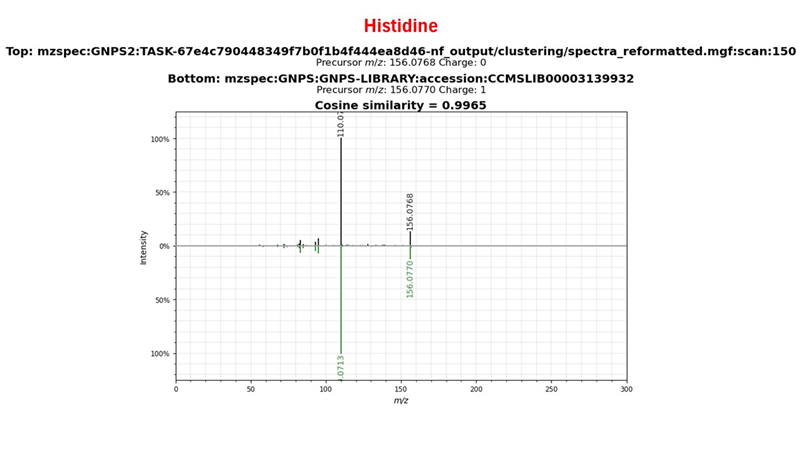


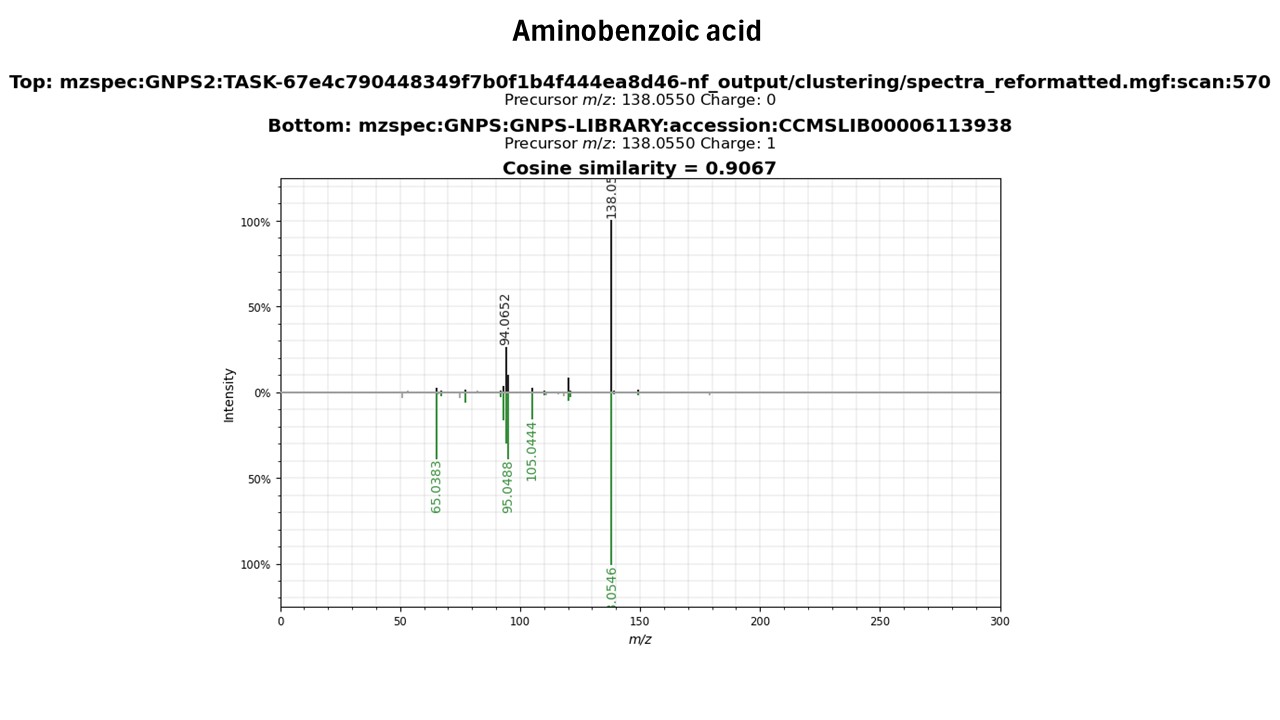

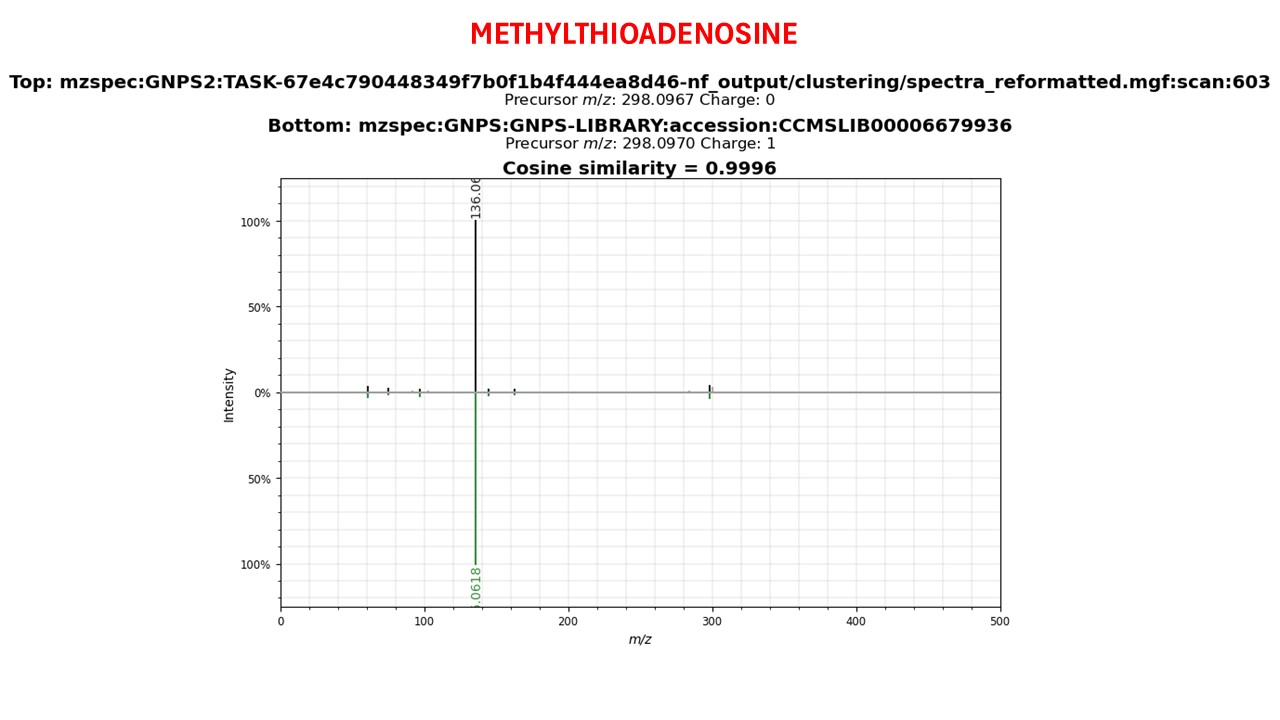

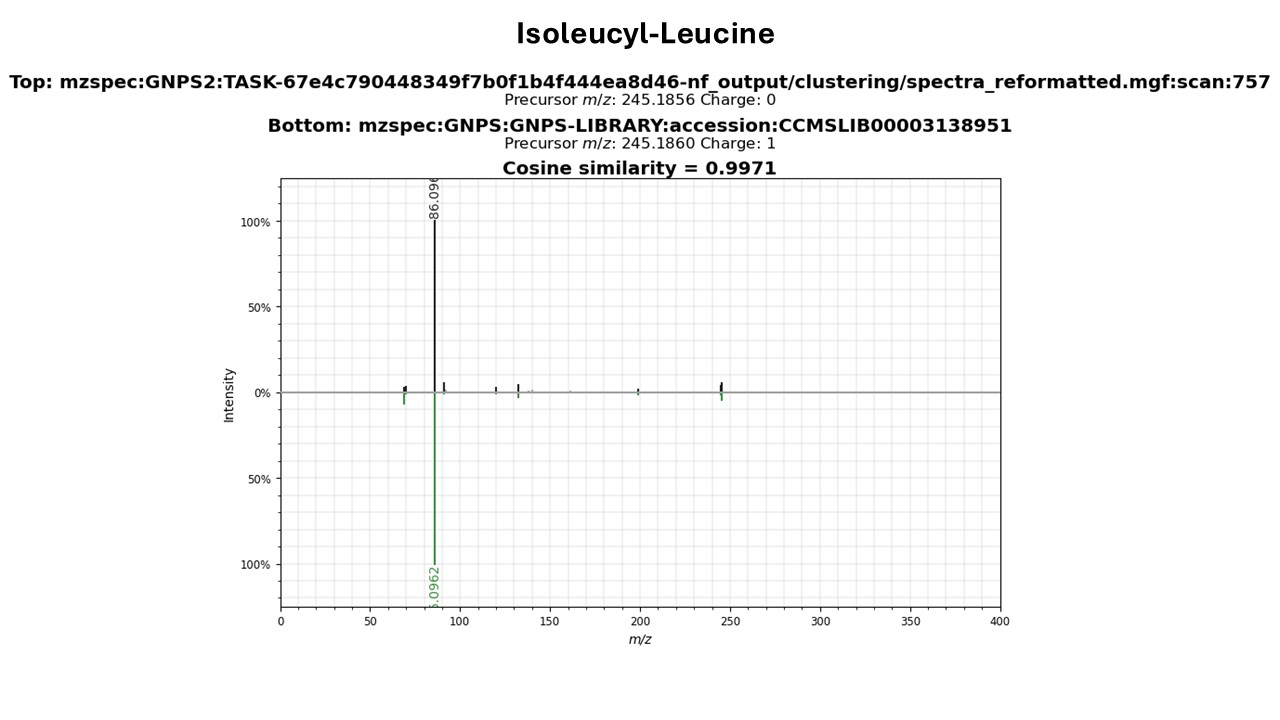

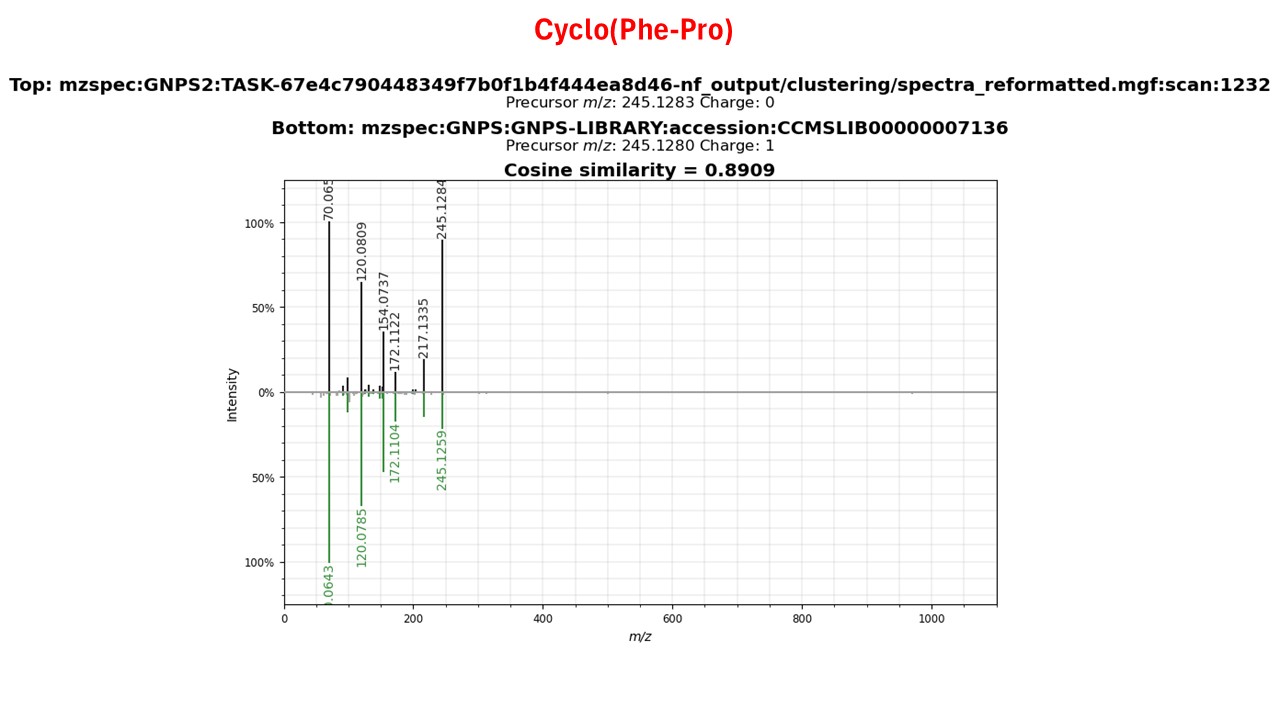

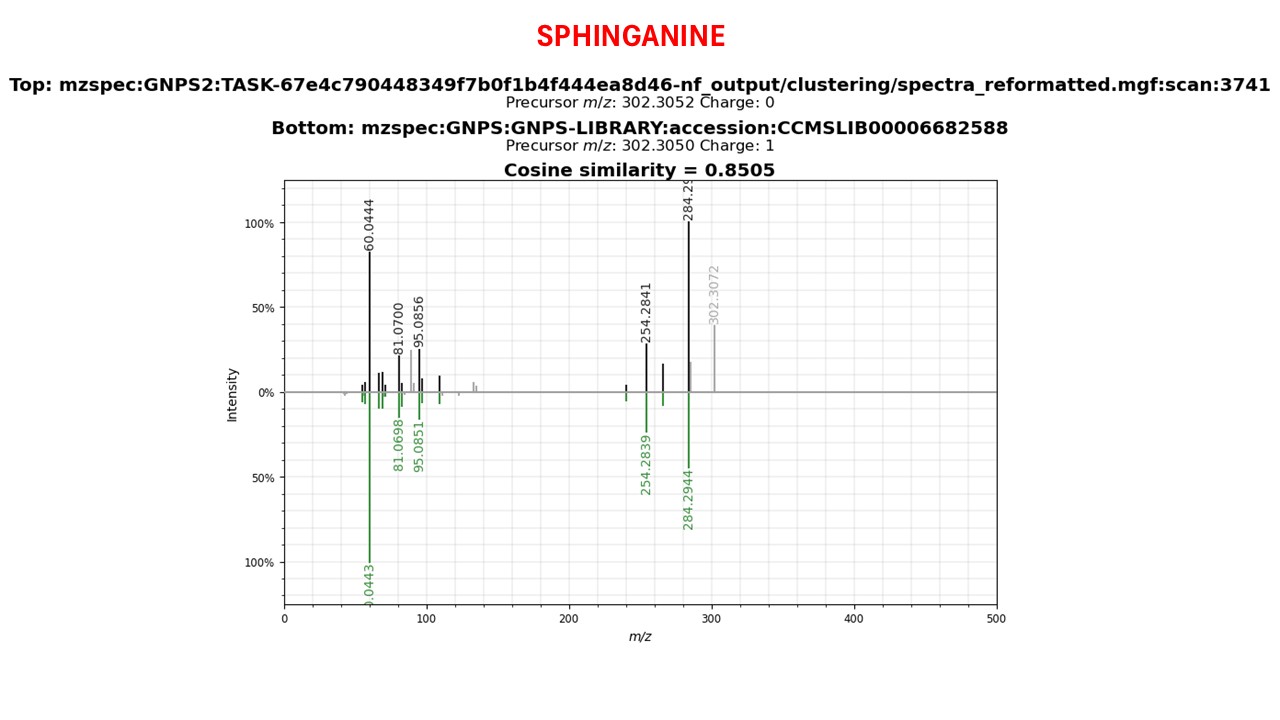

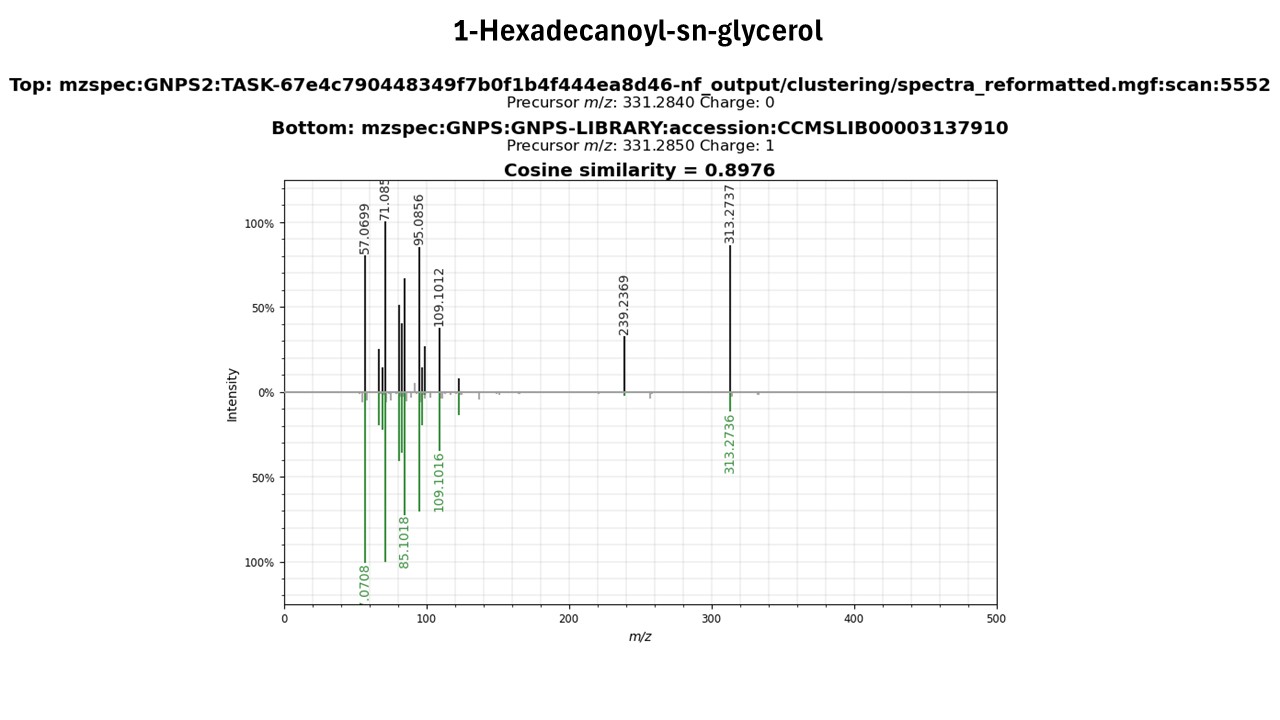


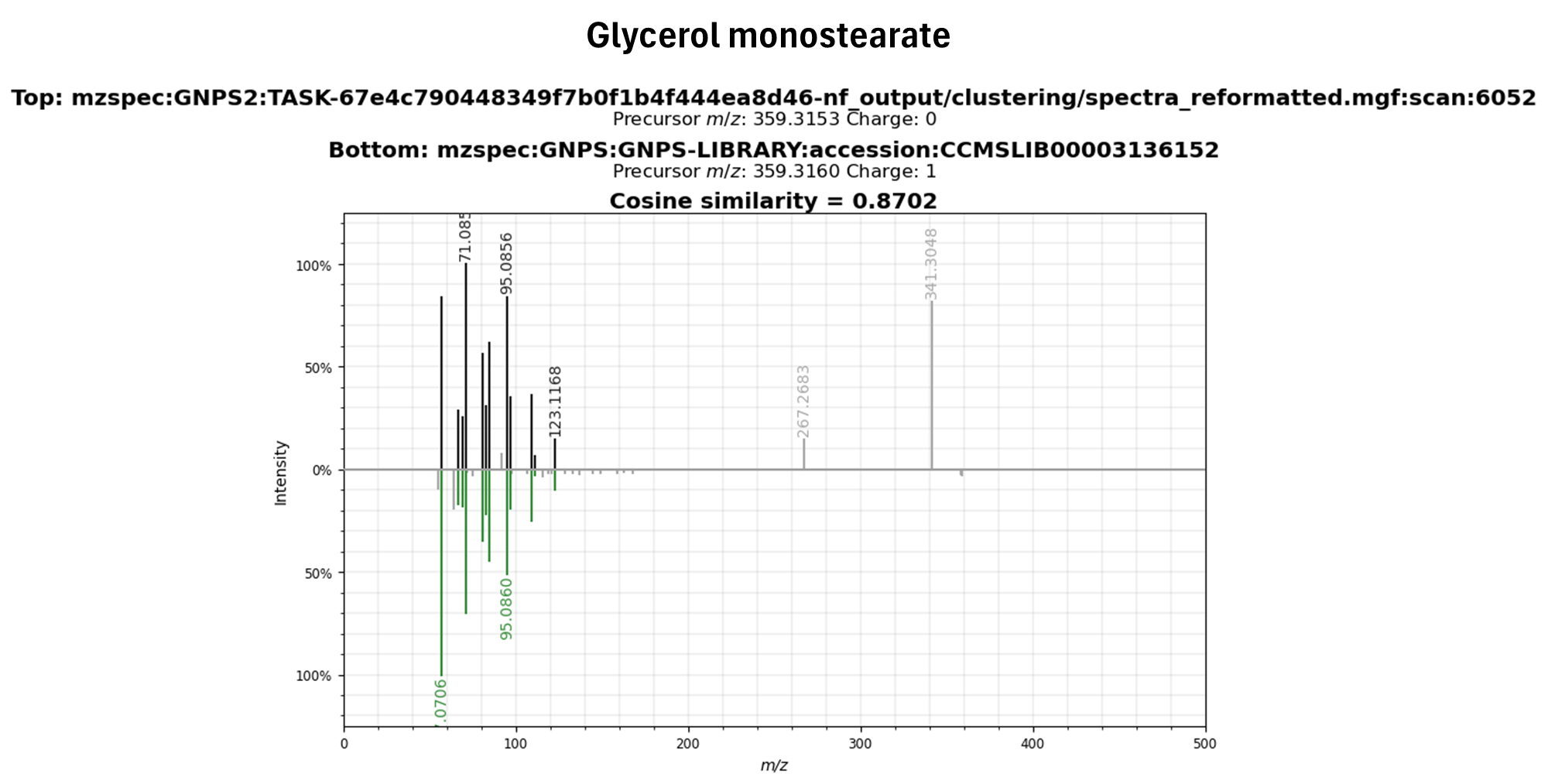

Supplement: Supplementary file 1 — 749Supporting Information 1. [file JOBM-66-e70149-s001.docx]
