# Supplementary material for: Metabolomic Analysis of Phytophthora parasitica Growth in the Presence of β‐sitosterol Indicates Adaptive Mechanisms Modulated by Sterols
Source: J Basic Microbiol. 2026 Feb 4;66(2):e70149. doi: 10.1002/jobm.70149 (PMC12873457; doi:10.1002/jobm.70149)

**Supporting Information 2**

Scatterplot matrix of the six components obtained from PLS-DA. Each point represents a metabolite (*row ID*), colored according to its mean VIP (Variable Importance in Projection) value, indicating the relative contribution of each metabolite to the separation among treatments. Components 1 through 6 are shown for all possible pairs, with diagonals displaying the density of each component.


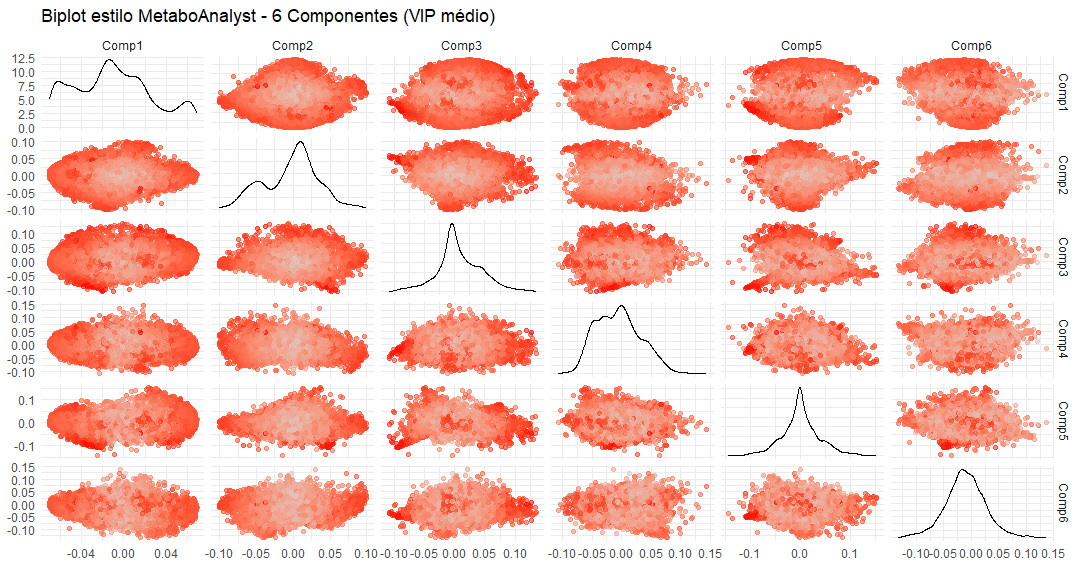

Supplement: Supplementary file 2 — 749Supporting Information 2. [file JOBM-66-e70149-s002.docx]
